# Ultrasonographic and cytologic assessments of follicular neoplasms of the thyroid: Predictive features differentiating follicular carcinoma from follicular adenoma

**DOI:** 10.1371/journal.pone.0271437

**Published:** 2022-07-21

**Authors:** Hye Shin Ahn, Hee Sung Kim, Min Ji Hong

**Affiliations:** 1 Department of Radiology, Chung-Ang University Hospital, Seoul, Korea; 2 Department of Pathology, Chung-Ang University Hospital, Seoul, Korea; Universidade do Porto Faculdade de Medicina, PORTUGAL

## Abstract

**Background:**

The preoperative diagnosis of follicular neoplasm of the thyroid is challenging due to difficulties in the assessment of capsular invasion. This study aimed to identify ultrasonographic (US) and cytopathologic features that are characteristic of follicular adenoma and carcinoma to aid in their differential diagnosis.

**Methods:**

A total of 98 surgically resected nodules diagnosed as follicular neoplasms between 2011 and 2012 were analyzed. US findings were reviewed according to the Korean Thyroid Imaging Reporting and Data System (K-TIRADS). Six cytologic features (high cellularity, abundant microfollicles, cell crowding/nuclear overlapping, isolated cells, homogeneous nuclei, abundant colloid) were reviewed quantitatively. The radiologic findings and quantification of cytologic features were correlated with final diagnoses.

**Results:**

In total, 70 (71.4%) and 28 (28.6%) of the nodules were follicular adenomas and follicular carcinomas, respectively. US findings of a heterogeneous echogenicity, speculated/ill-defined margin, and presence of calcifications were significantly associated with follicular carcinoma (p<0.05). Calcifications had a predilection for pericapsular areas than for stromal areas in follicular carcinomas, whereas their location was more varied in follicular adenomas. No cytologic feature was significantly different between follicular adenomas and carcinomas.

**Conclusion:**

Distinct from follicular adenomas, follicular carcinomas are characterized by heterogeneous echogenicity, speculated/ill-defined margin, and presence of calcifications on US. Thus, US findings can be helpful to differentiate between these two follicular neoplasms.

## Introduction

Thyroid nodules are very common, occurring in 50% of the adult population [1]. Approximately 20% of these nodules are diagnosed as follicular neoplasm on fine-needle aspiration (FNA) cytology [2]. Follicular neoplasm is a cytologic term that refers to both the benign proliferation of thyroid follicular cells in adenoma and the malignant proliferation in carcinoma [3,4]. Follicular adenomas are more common than follicular carcinomas and have no vascular or capsular invasion, but they otherwise share similar cytologic features with follicular carcinomas. In general, when a biopsy specimen of a thyroid nodule reveals a follicular neoplasm, approximately 80–90% of such lesions will be adenomas and 10–20% will be carcinomas [3,4]. Given these shared features, cytological differentiation between benign and malignant tumors is challenging [5]. Cytologic specimens cannot be histologically evaluated for capsular invasion. Moreover, some cases of frozen section specimen as well as core needle biopsy also cannot differentiated follicular adenoma between carcinoma because nodular capsule has not to be completely examined; thus, surgical excision such as lobectomy is recommended to aid in the diagnosis of follicular carcinoma and follicular adenoma, although this is highly invasive method [6,7].

Some studies have proposed ultrasonographic (US) findings and cytologic features to differentiate follicular carcinoma from adenoma [8–11]. Retrospective studies have reported the predictive usefulness of US findings for follicular carcinoma [8–10]. A recent pathologic study analyzed the utility of various cytologic features to increase the accuracy of cytologic diagnosis for follicular neoplasm [11]. However, no distinct findings characteristic of benign and malignant follicular neoplasms have been established. Thus, this study aimed to identify US findings and cytopathological features that can aid in the differential diagnosis between follicular adenoma and carcinoma.

## Materials and methods

### Study design and specimens

This retrospective study was approved by the appropriate Institutional Review Board at Chung-Ang University Hospital (2107-016-470) and informed consent was not required because it’s retrospective nature of the study.

A total of 120 patients with thyroid nodules who underwent surgical resection and diagnosed with follicular neoplasms between January 2011 and December 2012 were evaluated. Of these, 22 were excluded because of unavailable FNA result or FNA performed elsewhere (n = 15) and limited evaluation of US images due to outside US examination (n = 7). Finally, 98 nodules of 98 patients who initially diagnosed via FNA procedures and finally diagnosed by surgical resection were analyzed.

### US imaging and procedures

Thyroid US examination and procedures were performed by one of two experienced radiologists with 8 and 7 years of thyroid imaging experience, respectively. US examination was performed using high-resolution US equipment with a 12 MHz linear transducer (IU 22; Philips Ultrasound, Bothell, Washington, USA). US-guided FNA was performed using a conventional method, and at least two samples were taken per nodule. FNA was conducted using a 23-gauge needle attached to a 5-ml syringe. Successful sampling was achieved with numerous multidirectional passes through the nodule. Specimens were preserved in bottles with 95% ethanol for liquid-based cytological examination (Surepath). Repeat FNA was considered in cases of non-diagnostic cytological findings of nodules or atypia/follicular lesions of undetermined significance (AUS/FLUS).

### Radiologic and cytopathologic analyses

Radiologic and cytopathologic data of the 98 thyroid nodules were collected. Two experienced radiologists (HSA and MJH, who had 8 and 9 years of experience in performing thyroid US and interventional procedures, respectively) blinded to the FNA results or final diagnoses retrospectively reviewed the US images in consensus. The US features included size, composition, margin, echogenicity, orientation, and calcifications, and the nodules were categorized according to the Korean Thyroid Imaging Reporting and Data System (K-TIRADS) guideline developed by The Korean Society of Thyroid Radiology [12]. The thyroid nodules were categorized into four categories (benign, low suspicion, intermediate suspicion, and high suspicion) using the K-TIRADS, a malignancy risk-stratification system developed based on solidity, echogenicity, and suspicious US features in thyroid nodule. K-TIRADS 5(high suspicion) nodules include solid hypoechoic nodules with any suspicious US feature (microcalcification, non-parallel orientation, spiculated/microlobulated margin). K-TIRADS 4 (intermediate suspicion) nodules include solid hypoechoic nodules without suspicious US feature and partially cystic or iso-hyperechoic nodules with any suspicious US feature. K-TIRADS 3 (low suspicion) nodules include partially cystic or iso- or hyperechoic nodules with no suspicious US feature. K-TIRADS 2 (benign) nodules include pure cysts, partially cystic with comet tail artifacts, and spongiform nodules. In particular, the calcifications are categorized as microcalcifications (punctuate echogenic foci of 1 mm or less either with or without posterior shadowing), macrocalcifications (echogenic foci greater than 1 mm in size with posterior shadowing), and rim calcifications (peripheral curvilinear or eggshell calcification at the nodule margin).

**Fig 1:**
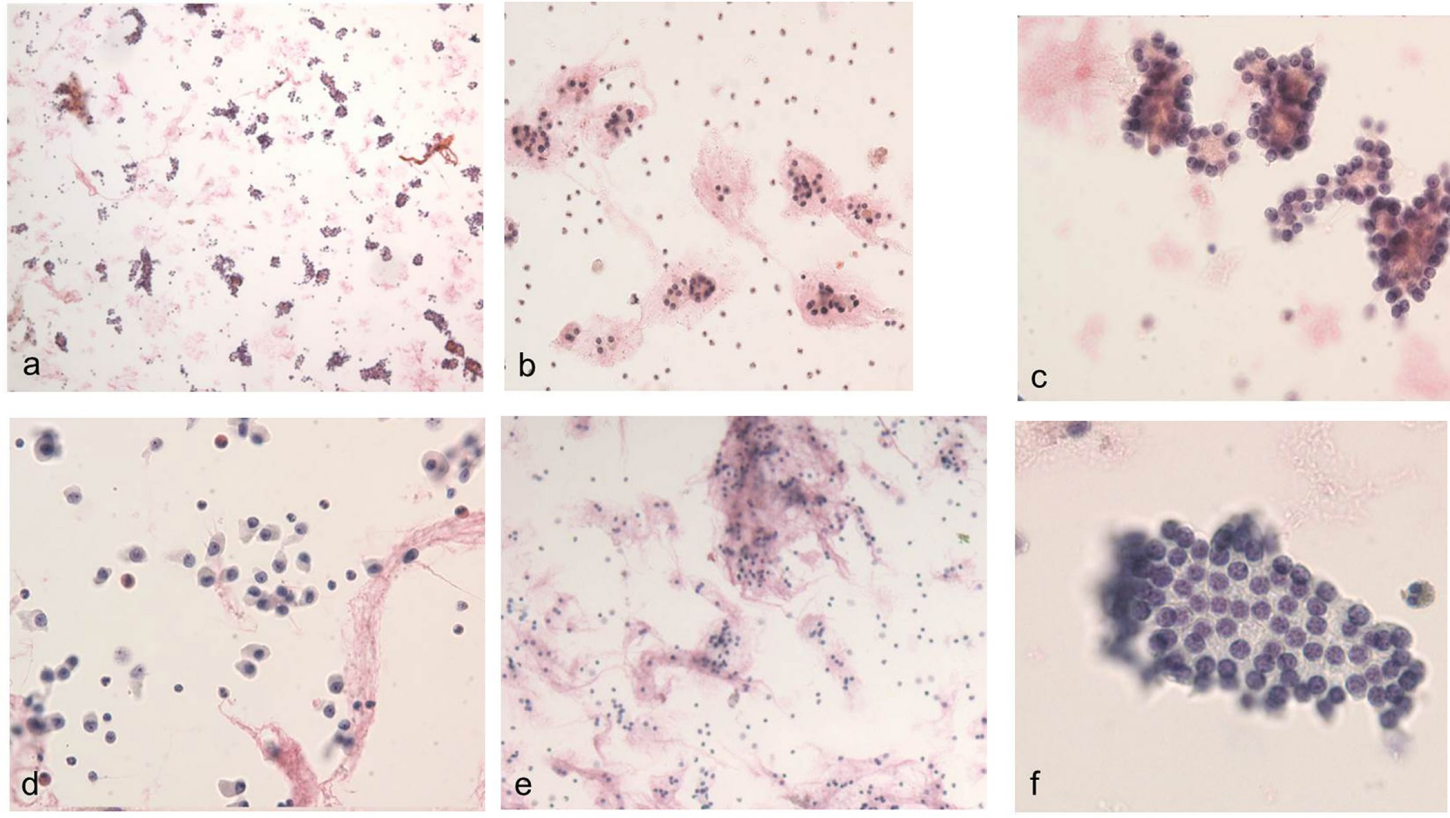
Cytologic features. (a) High cellularity (×40). (b) Abundant microfollicles (×100). (c) Cell crowding and nuclear overlapping (×400). (d) Isolated cells (×100). (e) Abundant colloids (×40). (f) Homogeneous nuclei (×400).

Cytopathologic findings were retrospectively reviewed by a thyroid pathologist with 20 years of experience (HSK). The interpretation of FNA was based on The Bethesda System for Reporting Thyroid Cytopathology [13]. FNA cytology results were classified as six categories of Bethesda System. In addition, the following six cytologic features were quantitatively reviewed: high cellularity, abundant microfollicles, cell crowding/nuclear overlapping, isolated cells, homogeneous nuclei, and abundant colloid. These were then classified into five categories based on previous literature by Yoo et al [11]: 0 (absent, 0%), 1, (minimal, <10%), 2 (mild, 10–40%), 3 (moderate, 41–70%), and 4 (marked, >70%). Each feature was summarized as a score and statistically compared with the Bethesda categorization and final diagnoses. Every cytologic feature is represented in Fig 1. For the cases in which multiple FNA (up to 2 times) was performed, the highest grade of cytologic diagnosis was selected.

### Reference standard and statistical analysis

The final surgical histopathologic findings of either total thyroidectomy or lobectomy were used as the reference standard (follicular adenoma vs. follicular carcinoma). The radiologic K-TIRADS findings and the quantified cytologic features were correlated with final diagnoses. Between-group comparisons of continuous variables, including age and nodule size, were conducted using the two sample t-test, while categorical variables were compared using the chi-square test and Fisher’s exact test. The quantified cytologic features were dichotomized as mild for 0, 1, and 2 and severe for 3 and 4 for chi-square or Fisher’s exact test. All statistical analyses were performed using commercially available statistical software (SPSS, version 20.0; SPSS, Chicago, IL). Significance was defined as p<0.05.

## Results

### Patient and nodule characteristics

The mean patient age was 50.8 ± 14.4 years, and most patients were female (n = 74 (75.5%); male, n = 24, 24.5%). Among the 98 thyroid nodules, 70 and 28 were follicular adenomas and follicular carcinomas, respectively. The lesion diameter on B-mode US ranged from 0.5 cm to 5.4 cm (mean: 1.9 cm). All diagnoses were surgically confirmed by total thyroidectomy (n = 62) or lobectomy (n = 36) (Table 1). Overall, 25 of the 98 nodules were subjected to multiple FNA. On the first FNA, 10 nodules were non-diagnostic (Bethesda I); 10 nodules, benign (Bethesda II); 33 nodules, AUS/FLUS (Bethesda III); 43 nodules, suspicious for follicular neoplasm/ follicular neoplasm (SFN/FN; Bethesda IV); and 2 nodules, papillary thyroid carcinoma (Bethesda VI). On the second FNA of 23 nodules, 4, 7, 4, and 8 nodules were categorized as non-diagnostic (Bethesda I), benign (Bethesda II), AUS/FLUS results (Bethesda III), and SFN/FN (Bethesda IV), respectively. On the third FNA of 2 nodules, 1 nodule was categorized as nondiagnostic (Bethesda I), while the other, benign (Bethesda II). The mean time span between first and second FNA was 93 days (range, 81 days to 105 days), between second and third FNA was 85 days (range, 77 days to 90 days). The mean time duration between final FNA of each nodules and surgical resection was 10 days (range, 7 days to 17 days).

**Table 1:** Clinicodemographic patient characteristics (n = 98).

Characteristics	Data
Age (years)	50.8 [range, 20-89]
Initial lesion size	1.9 [range, 0.5-5.4]
Pathologic type	
Follicular adenoma	70 (71.4)
Follicular carcinoma	28 (28.6)
FNA results	
First	73
Second	23
Third	2
Surgical method	
Total thyroidectomy	62 (63.3)
Lobectomy	36 (36.7)

Data are presented as the mean [range] or number of patients (percentage).

FNA, fine-needle aspiration.

### Radiographic and cytopathologic features

Among the six cytologic features evaluated, follicular adenomas and carcinomas shared features of high cellularity, abundant microfollicles, cell crowding/nuclear overlapping, and homogeneous nuclei and lack of isolated cells and abundant colloid. There were no significant differences in cytologic features between the follicular neoplasms (all p>0.05) (Table 2).

**Table 2:** Cytologic features correlated with the final diagnoses of thyroid nodules.

Characteristics	Follicular adenoma (n=70)	Follicular carcinoma (n=28)	P value
High cellularity, n (%)			0.618
Mild	19 (27.1)	6 (21.4)	
Severe	51 (72.9)	22 (78.6)	
Abundant microfollicles, n (%)			0.999
Mild	29 (41.4)	11 (39.3)	
Severe	41 (58.6)	17 (60.7)	
Cell crowding/nuclear overlapping, n (%)			0.578
Mild	14 (20.0)	4 (14.3)	
Severe	56 (80.0)	24 (85.7)	
Isolated cells, n (%)			0.598
Mild	53 (75.7)	23 (82.1)	
Severe	17 (24.3)	5 (17.9)	
Homogeneous nuclei, n (%)			0.628
Mild	20 (28.6)	10 (35.7)	
Severe	50 (71.4)	18 (64.3)	
Abundant colloid, n (%)			0.720
Mild	62 (88.6)	26 (92.9)	
Severe	8 (11.4)	2 (7.1)	

Data are presented as the number of patients (percentage).

Meanwhile, there were distinct sonographic features between the two follicular neoplasms. Heterogeneous echogenicity was more common in follicular carcinomas (28.6% vs. 11.4%, p = 0.042). Follicular carcinomas also more frequently showed an ill-defined margin (14.3% vs. 2.9%), whereas follicular adenomas more commonly had a smooth margin (97.1% vs. 82.1%) (p = 0.027). Calcifications were also more common in follicular carcinomas (35.7% vs. 14.3%, p = 0.048), with macrocalcification being the most frequent type (25%, 7/28). Macro- and rim calcifications were observed in 32.1% of follicular carcinomas, but only in 10.0% of follicular adenomas. Other US findings of nodule size, composition, echogenicity, orientation, shape, and K-TIRADS category were not significantly different between follicular carcinomas and adenomas. The sonographic features of follicular adenomas and carcinomas are presented in Table 3.

**Table 3:** US findings correlated with the final diagnoses of thyroid nodules.

Characteristics	Follicular adenoma (n=70)	Follicular carcinoma (n=28)	P value
Nodule size (cm), mean±SD	2.53 ± 1.25	3.37 ± 2.09	0.091
<1.0 cm	6 (8.6)	8 (28.6)	
≥1.0 cm	64 (91.4)	20 (71.4)	
Composition			0.593
Solid	50 (71.4)	18 (64.3)	
Predominantly solid	19 (27.1)	10 (35.7)	
Predominantly cystic	1 (1.4)	0 (0.0)	
Cystic	0 (0.0)	0 (0.0)	
Echogenicity			0.332
Marked hypoechoic	2 (2.9)	2 (7.1)	
Hypoechoic	24 (34.3)	6 (21.4)	
Isoechoic	44 (62.9)	20 (71.4)	
Hyperechoic	0 (0.0)	0 (0.0)	
Mixed echo			0.042
Homogeneous	62 (88.6)	20 (71.4)	
Heterogeneous	8 (11.4)	8 (28.6)	
Orientation			N/A
Parallel	70 (100.0)	28 (100.0)	
Non-parallel	0 (0.0)	0 (0.0)	
Margin			0.027
Smooth	68 (97.1)	23 (82.1)	
Spiculated	0 (0.0)	1 (3.6)	
Ill-defined	2 (2.9)	4 (14.3)	
Shape			0.714
Ovoid to round	69 (98.6)	28 (100.0)	
Irregular	1 (1.4)	0 (0.0)	
K-TIRADS			0.804
K-TIRADS 3	48 (68.6)	20 (71.4)	
K-TIRADS 4	21 (30.0)	8 (28.6)	
K-TIRADS 5	1 (1.4)	0 (0.0)	
Calcifications, n (%)			0.048
None	60 (85.7)	18 (64.3)	
Microcalcifications	3 (4.3)	1 (3.6)	
Macrocalcifications	6 (8.6)	7 (25.0)	
Rim calcifications	1 (1.4)	2 (7.1)	

Data are presented as the number of patients (percentage), unless otherwise specified.

K-TIRADS, Korean Thyroid Imaging Reporting and Data System.

**Fig 2:**
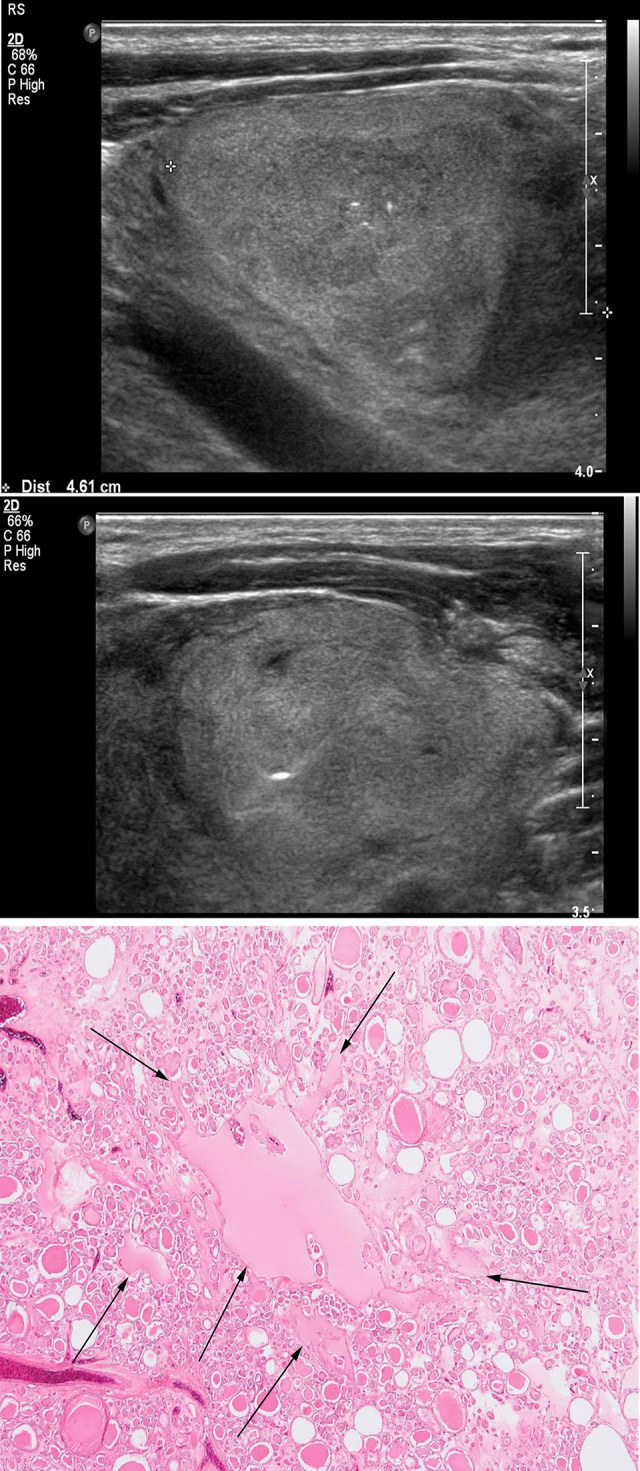
Images of a representative case of follicular adenoma. A 59-year-old man was diagnosed with follicular adenoma on US. (a) An US image showing a 4.6 cm–sized oval predominantly solid isoechoic nodule with internal microcalcification. (b) US-guided FNA is performed with a 21-gauge syringe, and the nodule is suspicious for a follicular neoplasm. (c) The surgical specimen shows microfollicular proliferation with one macrofollicle containing three pieces of intrafollicular calcification corresponding to the ultrasonographic image shown in Fig 2A (arrows). ×40 original magnification. Hematoxylin and eosin staining is used. US, ultrasonography; FNA, fine-needle aspiration.

**Fig 3:**
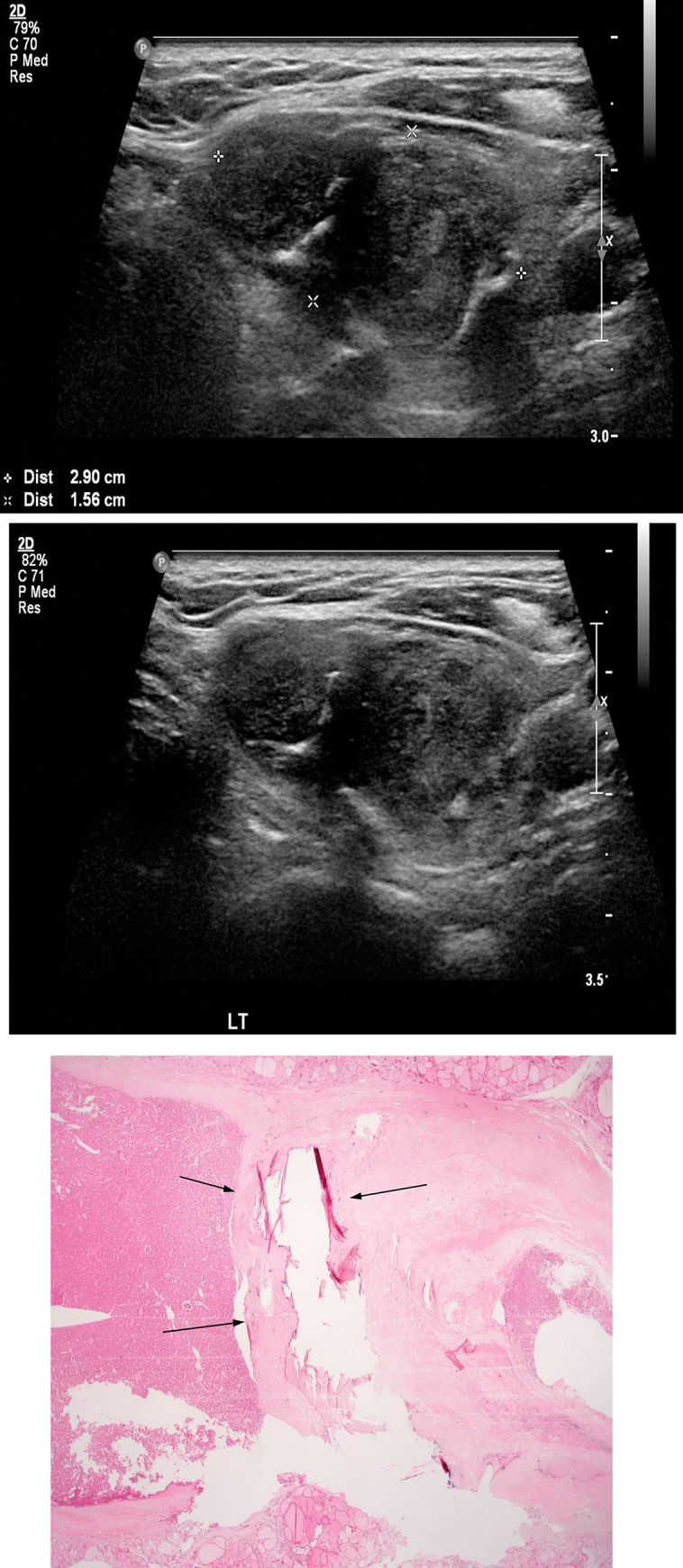
Images of a representative case of follicular adenoma. A 66-year-old woman was diagnosed with widely invasive follicular carcinoma. (a) An US image showing a 2.9 cm-sized heterogeneous ill-defined solid hypoechoic nodule with internal macrocalcification. (b) US-guided FNA is performed with a 21-gauge syringe, and the nodule appears to be a follicular lesion of undetermined significance. (c) The surgical specimen shows microfollicular proliferation with thick fibrous capsule with large pericapsular calcification corresponding to US image shown in Fig 3A (arrows). ×12.5 original magnification. Hematoxylin and eosin staining is used. US, ultrasonography; FNA, fine-needle aspiration.

The characteristics of the cases with nodule calcifications are shown in Table 4. With respect to location, calcifications were limited to the stromal (n = 3) or pericapsular (n = 7) area in follicular carcinomas. Meanwhile, it was more varied in follicular adenoma: follicular, 4 cases; stromal, 3 cases; and pericapsular, 3 cases. The calcifications were most frequently located in the follicle for follicular adenoma and in the pericapsule for follicular carcinoma (Figs 2 and 3).

**Table 4:** Characteristics of the patients with follicular neoplasm with calcifications.

Patient number	Age (years)	Sex	Nodule size (cm)	Calcification type on US	Calcification location	Final pathology
Follicular adenoma (n = 10)						
1	47	Female	1.7	Micro	Stroma	Follicular adenoma
2	40	Female	1.4	Macro	Stroma	Follicular adenoma
3	67	Female	1.0	Rim	Pericapsular	Follicular adenoma
4	65	Male	2.8	Macro	Pericapsular	Follicular adenoma
5	51	Female	0.9	Macro	Follicle	Follicular adenoma
6	59	Male	4.6	Micro	Follicle	Follicular adenoma
7	58	Male	2.2	Micro	Follicle	Follicular adenoma
8	34	Female	3.5	Macro	Follicle	Follicular adenoma
9	51	Female	1.3	Macro	Stroma	Follicular adenoma
10	35	Female	2.0	Macro	Pericapsular	Follicular adenoma
Follicular carcinoma (n = 10)						
11	58	Female	2.9	Macro	Stroma	Minimally invasive
12	65	Female	6.0	Macro	Pericapsular	Widely invasive
13	55	Male	3.6	Macro	Stroma	Minimally invasive
14	55	Female	2.1	Macro	Pericapsular	Minimally invasive
15	40	Female	0.5	Rim	Pericapsular	Minimally invasive
16	62	Female	3.1	Micro	Stroma	Minimally invasive
17	54	Female	8.9	Macro	Pericapsular	Widely invasive
18	66	Female	2.9	Macro	Pericapsular	Widely invasive
19	50	Female	4.0	Rim	Pericapsular	Widely invasive
20	37	Male	1.3	Macro	Pericapsular	Minimally invasive

US, ultrasonography.

## Discussion

Imaging features characteristic of benign and malignant follicular neoplasms have not been established. This study found that US features of heterogeneous echogenicity, spiculated/ill-defined margin, and presence of calcification are more common in follicular carcinoma than in follicular adenoma. Thus, they may be useful in the pathological diagnosis of follicular neoplasm before surgery.

Follicular neoplasms are challenging to classify cytologically because cytologic specimens cannot be histologically evaluated for capsular invasion. Therefore, recent guidelines recommend diagnostic surgery for patients with FN/SFN findings [7,14,15]. However, given that surgery is highly invasive, several studies have attempted to analyze cytologic features predictive of malignancy. Park et al. reported that atypism, which reveals higher anisocytosis, nuclear pleomorphism, coarse clumping of chromatin, and cellular overlapping, is more frequent in follicular carcinoma [16].

Another study reported that follicular neoplasm is characterized by the abundance of follicular epithelial cells, the presence of microfollicular structures, abundant cell crowding, abundant dispersed isolated cells, homogenous nuclear morphology, the lack of nuclear grooves, the lack of colloid material, and the lack of macrophages on cytology, making FNA a useful tool for differentiating follicular neoplasms [11]. However, in the current study, all six cytologic features reviewed (high cellularity, abundant microfollicles, cell crowding/nuclear overlapping, isolated cells, homogeneous nuclei, and abundant colloid) did not show significant differences between benign and malignant tumors. This is probably due to the limited evaluation of architectural histologic structures, including the nodule capsule.

Many retrospective studies have described the features of follicular neoplasm on US imaging. Although some of these studies reported that US had no diagnostic value in distinguishing follicular carcinoma from follicular adenoma [17,18], several studies have demonstrated its predictive capability for follicular carcinoma [19–24]. Zhang et al. reported that a US finding of heterogeneous echotexture is significantly associated with follicular carcinoma [19]. Another study by Shin et al. suggested a higher frequency of heterogeneous mulberry-like echotexture in invasive follicular thyroid carcinoma than in minimally invasive follicular carcinoma [20]. The heterogeneous echotexture may be due to tissue necrosis and/or hemorrhage within follicular carcinomas [19–21]. Another predictive feature is the lesion margin on US [22,23]. Pompili et al. proposed a scoring system for malignancy in cytologically diagnosed follicular lesions and reported that an irregular margin of the nodule is significantly correlated with malignancy [22]. Another recent study reported that spiculated margin on US is a predictive feature of follicular carcinoma [23].

Consistent findings were found in the current study. In addition, we identified that the presence of calcification within the nodule is significantly predictive of follicular carcinoma. While calcifications are more common in papillary thyroid carcinoma, they can be seen in follicular carcinomas. Grani et al. reported microcalcifications which suggestive of psammoma bodies are common in papillary thyroid cancer, whereas rim and coarse calcification can be found in both follicular and papillary thyroid cancer, probably due to necrosis and hemorrhage [25]. Our result in terms of calcifications is concordant with other previous studies [22–24]. In these studies, the presence of calcification accurately predicted malignancy, with odds ratios of 6.413–22.879 [22–24]. Shin et al. compared the US findings between widely and minimally invasive follicular thyroid carcinoma and found a higher frequency of calcifications in widely invasive follicular carcinoma [20]. Authors also reported that ring calcification is the most common type of calcification in invasive follicular carcinoma, and it arises from dystrophic calcifications deposited along intervening capsules. Zhang et al. a higher frequency of micro-/macro-calcifications and peripheral calcifications in follicular carcinoma than in follicular adenoma and suggested that these calcifications may be secondary to tissue necrosis, hemorrhage, or dystrophic changes [19].

In our study, we evaluated the US features of calcification and the location of calcification on the final pathology specimen. The results showed that macro- and rim calcifications were more common in follicular carcinoma (32.1%, 9/28), and 7 of 10 follicular carcinomas showed pericapsular location on final pathology. To the best of our knowledge, our study is the first to describe and differentiate the location of calcification on pathologic specimens in follicular neoplasms. The findings support the fact that US features of follicular neoplasms and the characteristics/locations of calcifications on thyroid nodules have a potential value to predict follicular carcinoma. Collectively, our data and those of previous studies support that US findings of spiculated/ill-defined margins, heterogeneity, and macro-/rim calcifications can help differentiate follicular carcinoma from follicular adenoma. In addition, the location of calcifications on final pathology may distinguish between follicular adenoma and carcinoma.

Our study has some limitations. The retrospective study design is associated with an unavoidable selection bias. The study design also prevented us from evaluating US findings in real time, which might have influenced the evaluation of the reviewers. In addition, surgery is not a common diagnostic modality for follicular neoplasms. Thus, our inclusion of only patients who have underwent surgery led to a small sample size. However, this bias is unavoidable because histopathology is necessary to establish the final diagnosis.

In conclusion, no cytologic feature is significantly different between follicular adenomas and carcinomas. However, US findings of a heterogeneous echogenicity, speculated/ill-defined margin, and presence of macro-/rim calcifications are characteristic of follicular carcinomas. Thus, these characteristics may be helpful in a preoperative diagnosis for a difficult case as determined by FNA in clinical suspected follicular neoplasm.

## Abbreviations

FNA: Fine-needle aspiration
US: Ultrasonography
